# *Cyphastrea salae*, a new species of hard coral from Lord Howe Island, Australia (Scleractinia, Merulinidae)

**DOI:** 10.3897/zookeys.662.11454

**Published:** 2017-03-21

**Authors:** Andrew H. Baird, Mia O. Hoogenboom, Danwei Huang

**Affiliations:** 1 ARC Centre of Excellence for Coral Reef Studies, James Cook University, Townsville, QLD, 4811, Australia; 2 College of Science and Engineering, James Cook University, Townsville, QLD, 4811, Australia; 3 Department of Biological Sciences & Tropical Marine Science Institute, National University of Singapore, Singapore 117543, Singapore

**Keywords:** Biodiversity, biogeography, cnidarian, coral reefs, phylogenetics

## Abstract

A new zooxanthellate reef-dwelling scleractinian coral species, *Cyphastrea salae* **sp. n.** (Scleractinia, Merulinidae), is described from Lord Howe Island Australia. The new species can be distinguished morphologically from the only other congeneric species on Lord Howe Island, *C. microphthalma*, by the number of primary septa (12 *vs.* 10) and the much taller corallites (mean ± SE: 1.0 ± 0.07 mm v 0.4 ± 0.04 mm). The relationship of *C. salae* to four of the other eleven currently accepted species in the genus was explored through analyses of nuclear (28S rDNA) and mitochondrial (noncoding intergenic region) gene sequences. *Cyphastrea salae* **sp. n.** forms a strongly supported clade that is distinct from a clade containing three species found commonly in Australia, *C. chalcidicum*, *C. serailia*, and *C. microphthalma*. One specimen was also found in the Solitary Islands, another high latitude location in south-eastern Australia. The discovery of a new species in the genus *Cyphastrea* on high latitude reefs in south-eastern Australia suggests that other new species might be found among more diverse genera represented here and that the scleractinian fauna of these isolated locations is more distinct than previously recognised.

## Introduction

The Indo-Pacific scleractinian genus *Cyphastrea* Milne Edwards & Haime, 1848, is one of the most distinctive genera in the recently revised family Merulinidae Verrill, 1865 (Huang et al. 2014). Unlike many other genera in the family Merulinidae, *Cyphastrea* has emerged with the same species composition (Huang et al. 2014), motivating more detailed work at the species level (e.g. Bouwmeester et al. 2015, Arrigoni et al. 2017). The World Register of Marine Species lists 24 nominal species in the genus *Cyphastrea*, of which 11 are considered valid (Hoeksema 2015). The genus was established by Milne Edwards and Haime (1848) for three species, *Cyphastrea microphthalma*, *C. savignyi* and *C. bottae*, distinguished by a compact coenosteum from species in the Indo-Pacific genera *Astrea* Lamark, 1801, *Plesiastrea*, Milne Edwards & Haime, 1848 and the West-Atlantic *Solenastrea* Milne Edwards & Haime, 1848. *Astrea* has been placed within Merulinidae, but is phylogenetically distant from *Cyphastrea* (Huang et al. 2014). *Plesiastrea*, represented by its type species *P. versipora* (Lamarck, 1816), is most closely related to the azooxanthallate corals *Trochocyathus efateensis* Cairns, 1999 and *Cyathelia axillaris* (Ellis & Solander, 1786), all of which are nested within clade XIV (*sensu* Fukami et al. 2008) and not in the Merulinidae (Kitahara et al. 2010, 2016, Huang et al. 2011, Huang and Roy 2013, 2015). Similarly, *Solenastrea* has been placed in clade XIII (*sensu* Fukami et al. 2008) along with several previously unaffiliated coral taxa such as *Oculina* Lamarck, 1816 (Fukami et al. 2008, Kitahara et al. 2010, 2016, Huang 2012). Nevertheless, *Cyphastrea* is the sister group to *Orbicella*, an Atlantic genus, and is therefore distinct among Indo-Pacific corals.

Lord Howe Island is a World Heritage-listed marine protected area in the Tasman Sea with highly distinctive marine fauna including nine endemic fish species (Francis 1993) and 47 endemic species of algae (MPA 2010). Over 100 scleractinian species have been recorded at Lord Howe Island, however, there is very little agreement as to which species are present. Veron and Done (1979) listed 61 species, Harriott et al. (1995) listed 59 species and Noreen (2010) listed 77 species, but only 37 of these species are common to all three studies. Such divergence in the taxonomic composition among different studies suggests that our understanding of this fauna remains incomplete. Lord Howe Island lies over 900 km south of the Great Barrier Reef (GBR) and coral populations are highly isolated (Ayre and Hughes 2004, Noreen et al. 2013). Such isolation creates potential for speciation, however, to date, no endemic scleractinian species have been described from Lord Howe Island. The Solitary Islands are a group of continental islands off the east coast of Australia and are of biogeographical interest due to the co-occurrence of subtropical species and tropical species at the limit of their southern range edges (Veron 1995; Mizerek et al. 2016). Approximately 70 corals species have been recorded in the Solitary Islands (Veron et al. 1974: Harriott et al. 1994).

The aim of this paper is to describe a new species, *Cyphastrea salae* sp n. and also to determine the number of species within the genus on Lord Howe Island. A total of three *Cyphastrea* spp. has been reported from Lord Howe Island: Veron (1974) listed only *C. serailia*, Veron and Done (1979) added *C. microphthalma*, and Harriott et al. (1995) added *C. chalcidium*.

## Materials and methods

**Sampling.** Specimens of *Cyphastrea* species were sampled using snorkel or SCUBA diving during several trips to Lord Howe Island and the Solitary Islands in eastern Australia (Appendix 2). Digital images of living colonies were taken, and then using a hammer and chisel, a sample of each colony was collected from which an approximate 1 cm^2^ subsample was preserved in absolute ethanol for molecular analysis. The rest of each specimen was placed in sodium hypochlorite for up to 48 h to remove all coral tissue, rinsed in fresh water and sun-dried.

The holotype and two paratypes of *Cyphastrea salae* sp. n. have been deposited at the Australian Museum (AM) in Sydney, Australia, along with four voucher specimens of *C. microphthalma* from the same area (Appendix 2).

**Imaging and measurements.** Images of skeletons were taken with a Canon G12. In addition, a small fragment of clean skeleton was chosen from representative specimens and mounted on a stub using double-sided carbon tape, sputter-coated with a 3nm layer of conductive gold-palladium (AuPd) film and examined using a Jeol JSM5410LVscanning electron microscope at the Advanced Analytical Centre at James Cook University.

To visualise the cross-sectional microstructure of the coralla, corallites were cut from each specimen transversely and then impregnated with epoxy and sectioned to a thickness of ~30 μm following Budd and Stolarski (2009, 2011). The resulting thin sections were examined under stereo or light microscope at magnifications of up to 100x. Images were taken of whole corallites as well as rapid accretion deposits and thickening deposits or fibres within the wall, septa and columella following Stolarski and Roniewicz (2001).

**Morphometric measurements.** The following variables were measured under a dissecting light microscope with an eyepiece micrometre at 20x magnification on five haphazardly selected corallites from each colony sample; corallite maximum diameter (maximum diameter from outer wall to outer wall), calyx maximum diameter (maximum diameter from the inner wall to inner wall), columella maximum diameter, corallite maximum height above the coenosteum (Appendix 2). In addition, the number of septal cycles and the number of primary septa were also recorded for each of the five replicate corallites per colony (Appendix 2). Finally, five replicate measures of polyp density were made for each colony sample by counting the number of corallites in five haphazardly placed 1-cm^2^ quadrats (Appendix 2). Differences in the mean of all continuous variables between the species where compared with *t*-tests with an adjusted alpha of 0.013.

**DNA extraction and molecular analyses.** Genomic DNA was extracted from each sample using the Qiagen DNeasy kit (Qiagen, Hilden, Germany) following the manufacturer’s instructions. Polymerase chain reaction (PCR) protocols followed Huang et al. (2011) to amplify two molecular markers, the nuclear 28S rDNA (Cuif et al. 2003) and mitochondrial noncoding intergenic region (IGR; between cytochrome oxidase subunit I and the formylmethionine transfer RNA gene) (Fukami et al. 2004). New sequences were deposited in GenBank under accession numbers KY630443-KY630465 (28S) and KY653212-KY653236 (IGR)

Sequences were organised into two separate data matrices using Mesquite 3.03 (Maddison and Maddison 2015), and supplemented with data of *C. chalcidicum*, *C. microphthalma* and *C. serailia* from previously-published analyses (Huang et al. 2011, 2014). A sample from Fiji identified as C. cf. decadia Moll & Best, 1984, and the outgroups *Paramontastraea salebrosa* (Nemenzo, 1955) and *Echinopora lamellosa* (Esper, 1795) (subclade XVII-I *sensu* Budd and Stolarski 2011; Huang et al. 2014) were also included. Alignments were carried out using the E-INS-i option in MAFFT 7.205 (Katoh et al. 2002, 2009, Katoh and Toh 2008, Katoh and Standley 2013) under default parameters.

Three optimality criteria were used to reconstruct phylogeny separately for each molecular marker. First, the maximum likelihood tree under the GTRGAMMA model was inferred using RAxML 8.0.9 (Stamatakis 2006, Stamatakis et al. 2008) and 50 random starting trees, with 1000 bootstrap replicates. Second, for Bayesian inference, we determined the most suitable model of molecular evolution using jModelTest 2.1.4 (Guindon and Gascuel 2003, Posada 2008, Darriba et al. 2012), testing for 24 models and choosing the best model based on the Akaike Information Criterion (AIC). Bayesian analyses were carried out in MrBayes 3.2.2 (Huelsenbeck and Ronquist 2001, Ronquist and Huelsenbeck 2003, Ronquist et al. 2012). Four Markov chains of 8 million iterations were generated in two runs, logging one tree per 100 generations. MCMC convergence among runs was assessed using Tracer 1.6 (Rambaut et al. 2014), and the first 10001 trees were discarded as burn-in. Third, maximum parsimony trees were inferred using tree searches performed in TNT 1.1 (Goloboff 1999, Nixon 1999, Goloboff et al. 2008) with 10000 random addition sequences, each employing 100 cycles of sectorial searches, ratcheting, drifting and tree fusing. Resampling was carried out through 1000 bootstrap replicates. Gaps were treated as missing data.

## Taxonomic account

### Cyphastrea

Taxon classificationAnimaliaScleractiniaMerulinidae

, Milne Edwards & Haime, 1848

#### Genus characters.

Corals are colonial by extracalicular budding. The coenosteum is typically spinose and corallites are small (< 4 mm diameter) with low relief (< 3 mm). Septa are in at most three cycles and paliform lobes are usually present. Septal teeth are multiaxial, low (< 0.3 mm) and closely set (< 0.3 mm). Walls are formed primarily by septotheca. Costa and septum centre clusters are weak, there is between 0.3–0.6 mm between costa clusters and < 0.3 mm between septum clusters.

### Cyphastrea salae sp. n.

Taxon classificationAnimaliaScleractiniaMerulinidae

http://zoobank.org/E9B09DB8-0F21-4721-983C-42E5FE9DA607

#### Material examined.

Holotype: Australian Museum AM 81_1530 South Flat, Lord Howe Island (LHI), Australia (-31.5611; 159.0741) 1 m depth. Paratypes: AM 81_1822 Malabar, LHI (-31.5115; 159.0575); AM 81_4749 Malabar West, LHI (-31.5118; 159.0508) and see Appendix 2.

#### Diagnosis.

*Cyphastrea salae* is found on Lord Howe Island and in the Solitary Islands, where it is the only *Cyphastrae* species with 12 primary septa.

#### Skeletal characteristics of the holotype.

**Figure 1.**
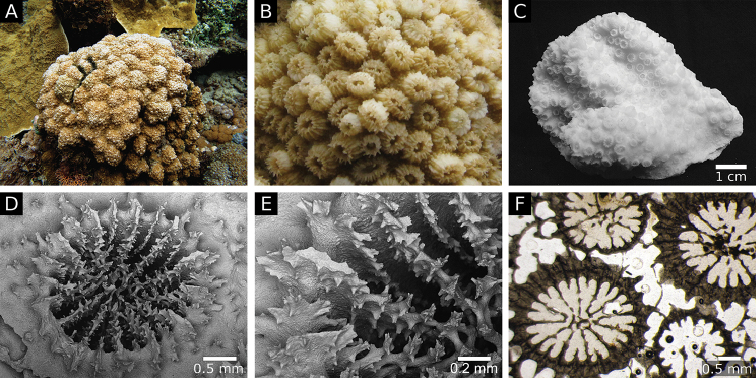
*Cyphastrea salae* sp. n. **A** habit photo of the holotype (G.18222) in situ **B** close up of holotype in situ **C** holotype **D** SEM of corallite **E** SEM of septa **F** transverse thin section.

The holotype is part of a hillocky colony approximately 50 cm width by 50 cm deep by 50 cm height of a tan colour (Fig. 1A). The most prominent feature of the species in the field are the exsert corallites (Figure 1B). The fragment of the holotype is 10 cm long by 5 cm wide and 4 cm thick (Fig. 1C). Most measured features were uniform: the maximum diameter of the corallites ranged from 2.5 to 2.8 mm, the maximum calyx diameter ranged from 2.0 to 2.4 mm and the maximum diameter of the columella ranged from 0.7 to 0.8 mm. All corallites had two septal cycles and 12 primary septa. In contrast, corallite height was more variable, ranging from 0.4 to 3.3 mm as was the density of corallites which ranged from 5 to 9 cm^-2^. The holotype has regular free septa and a compact columella (Figure 1D). Septal teeth with multiaxial tips are low (~0.2 mm in height) and narrowly spaced (0.1–0.2 mm), with 7–9 teeth per septum (Figure 1E). Strong pointed or club-shaped granules are scattered on the septal face (Figure 1E). The inter-area on the septa is smooth. Corallite walls are formed by dominant septotheca (Figure 1F). Thickening deposits are fibrous (Figure 1F). Costa and septum centre clusters are weak with approximately 0.3 mm between clusters in the costa and <0.2 mm in the septum. Medial lines are also weak (Figure 1F). Perpendicular crosses absent (Figure 1F). Columella centres are clustered (Figure 1F).

#### Instraspecific variation.

**Figure 2.**
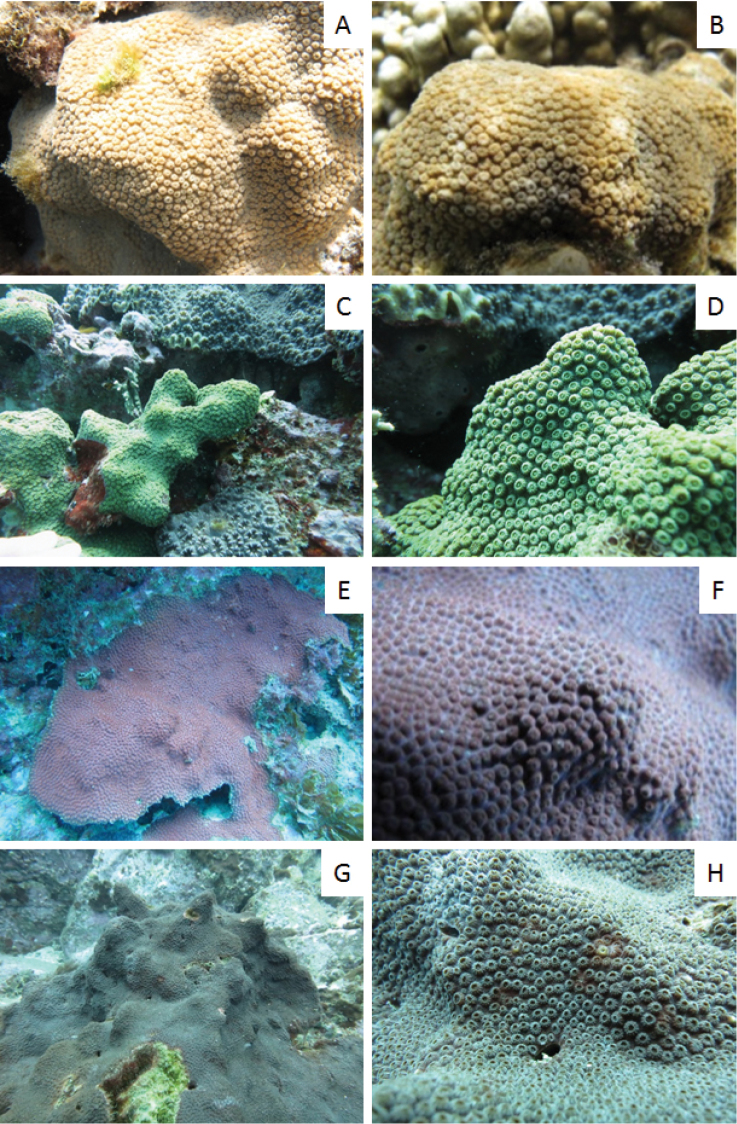
*Cyphastrea salae* in situ. **A, B** AM 81-1414 **C, D** AM 81_3953 **E, F** AM 81_1822 **G, H** AM 79_4749.

The most common morphology of colonies in the lagoon at Lord Howe Island is hillocky like the holotype, however, some coralla are massive (e.g. 81_1414; Figure 2A, B) or submassive (e.g. 81_3953; Figure 2C, D); and at depth the corolla can be encrusting (e.g. 81_1822; Fig. 2E, F and 79_4749; Figure 4G, H). The colour of the colony ranges from tan to green to blue. Corallite diameter ranges from 1.7 to 3.2 mm; calyx diameter ranges from 1.4 to 2.7 mm; columella diameter ranges from 0.6 to 1.0 mm; corallite height ranges from 0.4 to 3.5 mm; number of corallites per cm ranges from 5.0 to 14.0 cm^-2^; and the number of primary septa ranges from 10 in very small corallites to 17 in the largest (Table 1). Corallite density was noticeably reduced in specimens from greater depths on Lord Howe Island (Appendix 2; Figure 2E, F).

#### Comparison with *C. micropthalma*.

In general, there were few differences in the measured features of the corallites of *C. salae* and *C. microphthalma*. In particular, corallite and calyx diameter were remarkably similar between the two species (Table 1). Corallites were significantly taller (*t* = 3.43, p = 0.001) and the columella was larger (*t* = 7.12, p < 0.001) in *C. salae* (Table 1). In addition, corallites were significantly more crowded in *C. microphthalma* which had 11.4 corallites per cm^2^ compared with 9.6 per cm^2^, on average, for *C. salae* (*t* = -3.86, p < 0.001; Table 1).

**Table 1.** Summary statistics of morphological variables for *Cyphastrea salae* (n = 55) and *C. microphthalma* (n = 40). Measurements in mm.

Variable	Cyphastrea salae			Cyphastrea microphthalma		
	Mean (se)	Maximum	Minimum	Mean (se)	Maximum	Minimum
Corallite diameter	2.3 (0.05)	3.2	1.7	2.2 (0.05)	3.0	1.7
Calyx diameter	1.8 (0.04)	2.7	1.4	1.7 (0.04)	2.5	1.3
Columella diameter	0.6 (0.02)	1.0	0.4	0.5 (0.02)	1.0	0.4
Corallite height	1.0 (0.07)	3.5	0.4	0.4 (0.04)	0.8	0.0
Corallites per cm^2^	9.6 (0.30)	14.0	5.0	11.4 (0.35)	16.0	7.0
Number of primary septa	12.0 (0.15)	17	10	10.0 (0.12)	12	7

**Figure 3.**
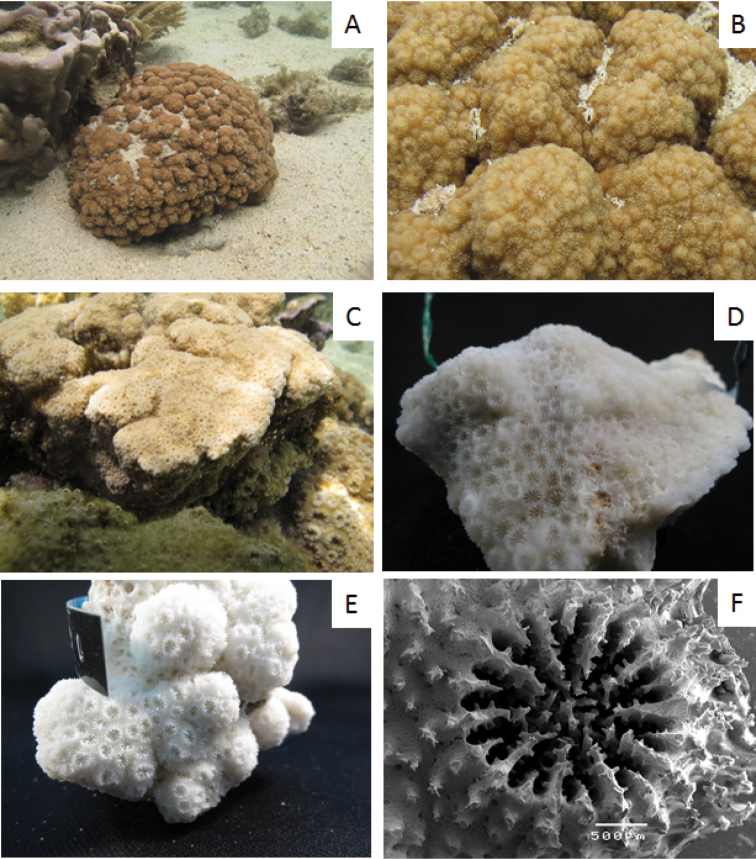
*Cyphastrea microphthalma*. **A, B** AM 81-1612 **C** AM 81_1681 **D** AM 81-1612 **E** AM 81-1660 **F** AM 81-1612.

The two species are difficult to distinguish in the field based on gross morphology. *Cyphastrea microphthalma* most frequently forms hillocky colonies (Figure 3 A, B), however, it can also occasionally form massive colonies (Figure 3C). Nonetheless, the species can readily be distinguished in the field and the lab on the basis of the number of primary septa which is generally 12 in *C. salae* (Figure 1C, D) *vs.* 10 in *C. microphthalama* (Figure 3D–F). Nonetheless, the modal number of septa in the five corallites counted correctly identified the molecular clade identity in 100% of colonies (Appendix 2).

#### Phylogenetic analysis.

**Figure 4.**
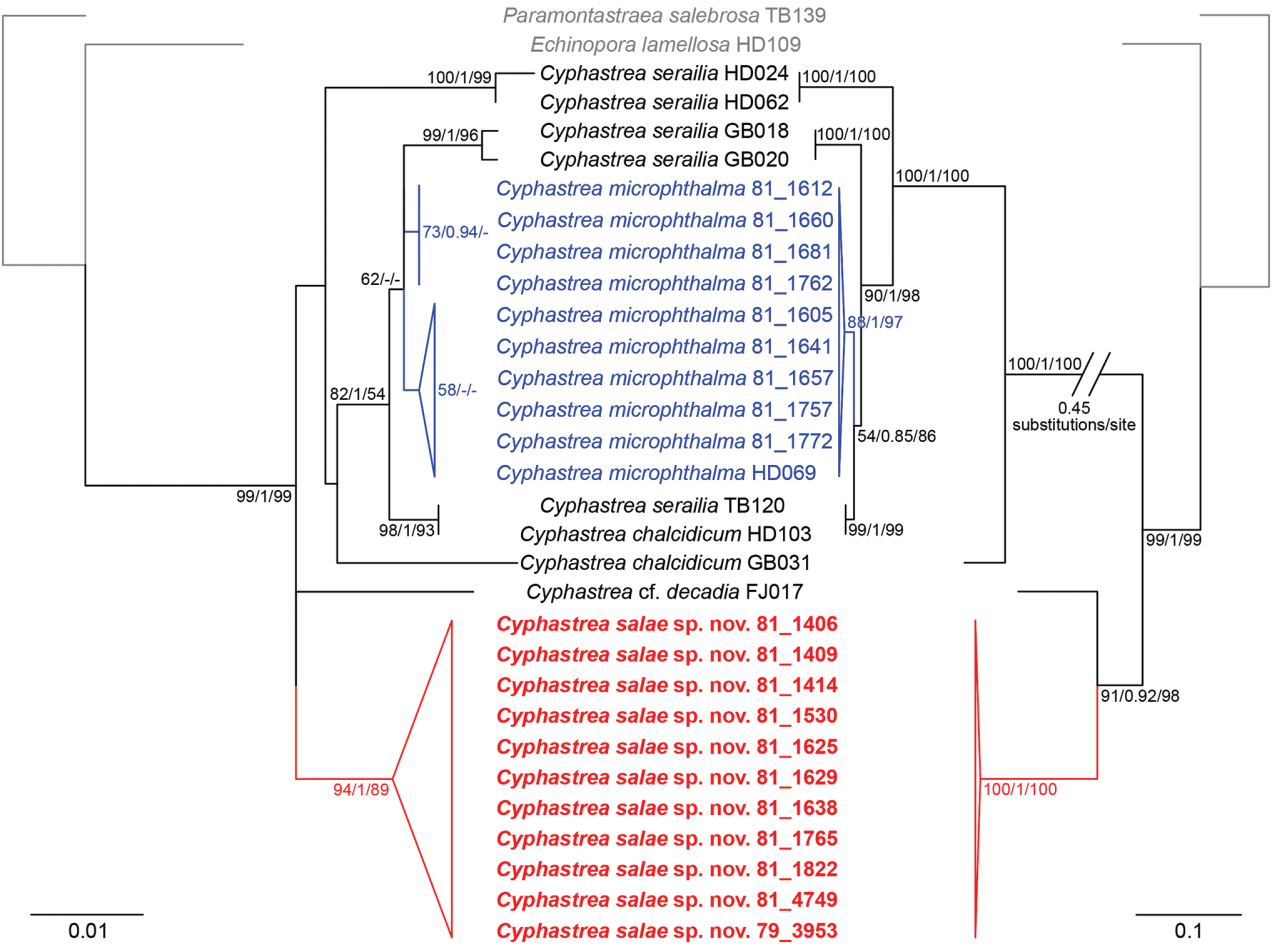
Maximum likelihood phylogenies based on the nuclear 28S rDNA (left) and mitochondrial noncoding intergenic region (right). Taxa in grey are outgroups. *Cyphastrea salae* sp. n. and *C. microphthalma* in red and blue respectively. Numbers adjacent to each branch represent support values (maximum likelihood bootstrap ≥ 50; Bayesian posterior probability ≥ 0.85; maximum parsimony bootstrap ≥ 50).

Molecular analyses revealed a monophyletic *Cyphastrea* with two major clades (Figure 4). One clade contained new sequences from all the *C. microphthalma* specimens collected from Lord Howe Island, nested within GenBank sequences of *C. microphthalma* from Singapore and the *C. chalcidicium*/*serailia* complex. The second clade comprised all of the sequences from *C. salae* from Lord Howe Island and the Solitary Islands, as well as C. cf. decadia from Fiji. The first clade was generally unsupported under all optimality criteria for 28S rDNA, but descendent nodes grouping *C. microphthalma* from Lord Howe Island with other *C. chalcidicum/serailia* and *C. microphthalma* sequences had moderate to high supports. The second clade containing *C. salae* was well-supported under all analyses, and is, although the sister relationship to the branching C. cf. decadia is only strongly supported by IGR (Figure 4).

*Cyphastrea salae* and *C. microphthalma* originated from two distinct lineages. *Cyphastrea salae* formed a well-supported monophyletic group under all analyses, and is sister group to the branching C. cf. decadia (Figure 4). All the IGR sequences of *C. microphthalma* clustered strongly as a clade, including the representative from Singapore, but the group is split weakly into two with the 28S rDNA marker. The relationships between *C. microphthalma* and its closely related congeners remained unresolved due to low support by the less variable 28S rDNA (Figure 4).

#### Etymology.

The species is named after Dr Sally Keith and Ms Sallyann Gudge, who have both made significant contributions to understanding and protecting the coral reefs of Lord Howe Island.

#### Distribution and frequency.

*Cyphastrea salae* is common in the lagoon at Lord Howe Island where it commonly co-occurs with *C. microphthalma*. It has been recorded to 18 m depth outside the lagoon. One colony has been positively identified using the molecular markers from Solitary Islands, where it is sympatric with *C. microphthalma*. *Cyphastrea salae* has yet to be recorded in extensive sampling on the Great Barrier Reef and no sequences are present in GenBank.

## Discussion

*Cyphastrea salae* sp. n. is genetically distinct from all other *Cyphastrea* sampled to date, forming a well-supported clade possibly closely related to C. cf. decadia from Fiji. All 18 specimens of *Cyphastrea* from Lord Howe Island fell into one of two clades suggesting that there are only two species on Lord Howe Island. *Cyphastrea salae* is also morphologically distinct from *C. microphthalma* on Lord Howe Island; in particular, the corallites are taller and the modal number of septa is 12 in *C. salae vs.* 10 in *C. microphthalma* (Table 1).

Amidst the polyphyly of *C. chalcidicum* and the poor support of 28S rDNA for *C. microphthalma*, the strong cohesion of *C. salae* is remarkable. The recovery of the massive *C. salae* as sister group to a branching specimen identified as C. cf. decadia from Fiji further highlights recent work demonstrating that morphology is a poor indicator of phylogeny (Arrigoni et al. 2016a, 2016b, Terraneo et al. 2016). *Cyphastrea* is a poorly sampled genus, with only six of the 11 currently accepted species sequenced for phylogenetic studies to date. More work is needed in this *C. salae* + C. cf. decadia clade, which is recovered here for the first time. We expect more comprehensive analyses of this distinct clade to reveal yet more interesting evolutionary patterns.

The number of primary septa is a highly reliable character for distinguishing between these two species on Lord Howe Island. The modal number of primary septa in a random sample of five polyps per colony correctly places all colonies in each molecular clade. Following Veron (2000), *C. salae* keys to *C. serailia* on the basis of its massive growth form, equal costae and 12 primary septa that are not irregularly exsert. *Cyphastrea salae* can be distinguished from *C. serailia* by the fact that corallites are mostly one size *vs.* mixed in *C. serailia*.

The spawning times also indicate a potential reproductive barrier to cross-fertilization between the species on Lord Howe Island. Five colonies of *C. microphthalma* released bundles of sperm and eggs on 19 January 2012 at 21:10 h and one colony of *C. salae* sp. n. released bundles of sperm and eggs on 20 January at 20:30 h (Baird et al. 2015).

The global distribution of *C. salae* remains uncertain. One specimen has been identified in collections from the Solitary Islands (Table 1; Figure 2G, H) and none to date from extensive sampling on the Great Barrier Reef or other sequences listed at GenBank. It is highly likely, therefore, that *C. salae* is a high-latitude endemic. When considered with the recent discovery of another high-latitude endemic, *Pocillopora aliciae* (Schmidt-Roach et al., 2012), this suggests that sub-tropical locations have a much higher level of endemism than previously recognised. This finding greatly increases the recognition of the conservation significance of high-latitude coral reef regions.

## Supplementary Material

XML Treatment for Cyphastrea

XML Treatment for Cyphastrea salae

## Appendix 1

**Table S1.** Specimens sampled. All specimens were collected by AHB.

**Specimen**	**Species**	**Location**	**Site**	**Latitude / Longitude**	**Depth_m**	**Date**	**Type**	**Aust_Mus_accession number**
81_1605	*Cyphastrea microphthalma*	Lord Howe Island	North Bay	-31.5214, 159.0468	1	12/01/2012		
81_1612	*Cyphastrea microphthalma*	Lord Howe Island	North Bay	-31.5214, 159.0468	1	21/01/2012	voucher	G.18226
81_1641	*Cyphastrea microphthalma*	Lord Howe Island	North Bay	-31.5214, 159.0468	1	21/01/2012		
81_1657	*Cyphastrea microphthalma*	Lord Howe Island	North Bay	-31.5214, 159.0468	1	21/01/2012		
81_1660	*Cyphastrea microphthalma*	Lord Howe Island	North Bay	-31.5214, 159.0468	1	21/01/2012	voucher	G.18225
81_1681	*Cyphastrea microphthalma*	Lord Howe Island	North Bay	-31.5214, 159.0468	1	21/01/2012		
81_1757	*Cyphastrea microphthalma*	Lord Howe Island	Horseshoe Reef	-31.5371, 159.0649	1	22/01/2012	voucher	G.18227
81_1762	*Cyphastrea microphthalma*	Lord Howe Island	Horseshoe Reef	-31.5371, 159.0649	1	22/01/2012		
81_1772	*Cyphastrea microphthalma*	Lord Howe Island	Horseshoe Reef	-31.5371, 159.0649	1	22/01/2012		
81_1530	*Cyphastrea salae*	Lord Howe Island	South Flat	-31.5611, 159.0741	1	20/01/2012	holotype	G.18222
81_1625	*Cyphastrea salae*	Lord Howe Island	North Bay	-31.5214, 159.0468	1	21/01/2012		
81_1629	*Cyphastrea salae*	Lord Howe Island	North Bay	-31.5214, 159.0468	1	21/01/2012		
81_1638	*Cyphastrea salae*	Lord Howe Island	North Bay	-31.5214, 159.0468	1	21/01/2012		
81_1765	*Cyphastrea salae*	Lord Howe Island	Horseshoe Reef	-31.5371, 159.0649	1	22/01/2012		
81_1822	*Cyphastrea salae*	Lord Howe Island	Malabar	-31.5115, 159.0575	8	22/01/2012	paratype	G.18223
81_4749	*Cyphastrea salae*	Lord Howe Island	Malabar West	-31.5118, 159.0508	10	20/03/2013	paratype	G.18224
81_1406	*Cyphastrea salae*	Lord Howe Island	North Bay	-31.5214, 159.0468	1	21/01/2012		
81_1409	*Cyphastrea salae*	Lord Howe Island	North Bay	-31.5214, 159.0468	1	21/01/2012		
81_1414	*Cyphastrea salae*	Lord Howe Island	North Bay	-31.5214, 159.0468	1	21/01/2012		
79_3953	*Cyphastrea salae*	South Solitary Island	Buchanen’s Wall	-30.2063, 153.2657	8	6/03/2013	voucher	G.18221

## Appendix 2

**Table S2.** Trait matrix

colony	species_molecular	corallite_number	corallite_diameter mm	calyx_diameter mm	columella_diameter mm	corallite_height	speta_no_primary	corallitescm-2	septal_cycles
81_1605	*Cyphastrea microphthalma*	1	2.4	1.7	0.5	0.5	10	11	2
81_1605	*Cyphastrea microphthalma*	2	2.0	1.4	0.5	0.8	10	9	2
81_1605	*Cyphastrea microphthalma*	3	2.0	1.7	0.6	0.1	10	12	2
81_1605	*Cyphastrea microphthalma*	4	2.0	1.7	0.6	0.3	10	10	2
81_1605	*Cyphastrea microphthalma*	5	2.3	1.5	0.5	0.4	10	11	2
81_1612	*Cyphastrea microphthalma*	1	2.2	2.0	0.9	0.5	11	14	2
81_1612	*Cyphastrea microphthalma*	2	2.5	2.0	0.6	0.1	10	12	2
81_1612	*Cyphastrea microphthalma*	3	2.4	2.0	0.6	0.4	10	14	2
81_1612	*Cyphastrea microphthalma*	4	3.0	2.5	1.0	0.7	18	12	3
81_1612	*Cyphastrea microphthalma*	5	2.5	1.9	0.6	0.8	10	11	2
81_1657	*Cyphastrea microphthalma*	1	2.5	1.7	0.6	0.8	10	10	2
81_1657	*Cyphastrea microphthalma*	2	2.2	1.8	0.5	0.4	10	10	2
81_1657	*Cyphastrea microphthalma*	3	2.5	1.7	0.5	0.5	11	8	2
81_1657	*Cyphastrea microphthalma*	4	2.0	1.7	0.5	0.0	10	8	2
81_1657	*Cyphastrea microphthalma*	5	2.2	1.7	0.5	0.3	10	10	2
81_1660	*Cyphastrea microphthalma*	1	2.0	1.5	0.4	0.3	9	10	2
81_1660	*Cyphastrea microphthalma*	2	2.2	1.7	0.4	0.3	10	12	2
81_1660	*Cyphastrea microphthalma*	3	2.2	1.5	0.6	0.4	10	10	2
81_1660	*Cyphastrea microphthalma*	4	2.2	1.8	0.5	0.3	10	7	2
81_1660	*Cyphastrea microphthalma*	5	2.0	1.6	0.5	0.2	7	13	2
81_1681	*Cyphastrea microphthalma*	1	1.7	1.3	0.5	0.0	10	16	2
81_1681	*Cyphastrea microphthalma*	2	1.7	1.3	0.4	0.2	10	15	2
81_1681	*Cyphastrea microphthalma*	3	1.7	1.5	0.5	0.0	9	11	2
81_1681	*Cyphastrea microphthalma*	4	1.7	1.3	0.4	0.2	9	13	2
81_1681	*Cyphastrea microphthalma*	5	2.4	1.5	0.5	0.2	10	12	2
81_1757	*Cyphastrea microphthalma*	1	2.6	1.9	0.5	0.5	12	10	2
81_1757	*Cyphastrea microphthalma*	2	2.5	2.1	0.5	0.0	11	15	2
81_1757	*Cyphastrea microphthalma*	3	2.4	1.9	0.4	0.2	10	16	2
81_1757	*Cyphastrea microphthalma*	4	2.5	2.2	0.6	0.3	10	11	2
81_1757	*Cyphastrea microphthalma*	5	2.2	1.7	0.4	0.5	10	13	2
81_1762	*Cyphastrea microphthalma*	1	2.5	1.8	0.5	0.4	10	8	2
81_1762	*Cyphastrea microphthalma*	2	2.2	1.8	0.4	0.3	10	14	2
81_1762	*Cyphastrea microphthalma*	3	1.7	1.4	0.4	0.2	10	10	2
81_1762	*Cyphastrea microphthalma*	4	2.4	1.8	0.4	0.8	11	10	2
81_1762	*Cyphastrea microphthalma*	5	2.4	1.7	0.5	0.8	10	13	2
81_1772	*Cyphastrea microphthalma*	1	2.3	2.0	0.6	0.3	11	10	2
81_1772	*Cyphastrea microphthalma*	2	2.4	1.7	0.5	0.6	9	10	2
81_1772	*Cyphastrea microphthalma*	3	2.5	1.8	0.6	0.3	10	9	2
81_1772	*Cyphastrea microphthalma*	4	2.3	1.8	0.6	0.2	10	13	2
81_1772	*Cyphastrea microphthalma*	5	2.4	1.9	0.6	0.3	10	12	2
79_3953	*Cyphastrea salae*	1	2.2	1.8	0.7	1.2	11	14	2
79_3953	*Cyphastrea salae*	2	2.5	2.0	0.7	1.0	12	10	2
79_3953	*Cyphastrea salae*	3	3.0	2.4	0.8	1.0	15	11	3
79_3953	*Cyphastrea salae*	4	2.2	1.8	0.5	1.3	12	11	2
79_3953	*Cyphastrea salae*	5	2.3	1.7	0.7	0.8	12	14	2
81_1406	*Cyphastrea salae*	1	2.7	2.0	0.7	0.8	13	9	2
81_1406	*Cyphastrea salae*	2	2.8	2.1	0.8	1.2	12	7	2
81_1406	*Cyphastrea salae*	3	2.5	2.2	0.8	1.7	12	10	2
81_1406	*Cyphastrea salae*	4	2.3	1.5	0.6	0.6	11	8	2
81_1406	*Cyphastrea salae*	5	2.2	2.0	0.7	0.8	11	7	2
81_1409	*Cyphastrea salae*	1	3.2	2.7	0.7	0.8	17	10	3
81_1409	*Cyphastrea salae*	2	2.4	1.8	0.5	1.5	11	9	2
81_1409	*Cyphastrea salae*	3	2.8	2.3	0.7	0.5	16	14	3
81_1409	*Cyphastrea salae*	4	2.4	1.4	0.6	1.6	11	8	2
81_1409	*Cyphastrea salae*	5	2.4	1.4	0.6	1.2	11	11	2
81_1414	*Cyphastrea salae*	1	2.4	1.7	0.6	0.5	11	10	2
81_1414	*Cyphastrea salae*	2	2.3	1.7	0.5	0.6	12	13	2
81_1414	*Cyphastrea salae*	3	2.2	1.8	0.7	0.6	12	9	2
81_1414	*Cyphastrea salae*	4	2.2	1.7	0.5	0.8	12	10	2
81_1414	*Cyphastrea salae*	5	2.1	1.5	0.5	0.8	11	12	2
81_1530	*Cyphastrea salae*	1	2.7	2.3	0.8	0.4	12	5	2
81_1530	*Cyphastrea salae*	2	2.7	2.3	0.8	1.5	12	7	2
81_1530	*Cyphastrea salae*	3	2.5	2.0	0.7	1.2	12	9	2
81_1530	*Cyphastrea salae*	4	2.8	2.4	0.7	3.5	12	5	2
81_1530	*Cyphastrea salae*	5	2.8	2.0	0.7	2.0	12	8	2
81_1597	*Cyphastrea salae*	1	3.0	2.3	0.8	0.7	12	9	2
81_1597	*Cyphastrea salae*	2	2.8	2.5	0.8	1.2	12	8	2
81_1597	*Cyphastrea salae*	3	3.2	2.7	0.8	0.6	15	7	2
81_1597	*Cyphastrea salae*	4	2.7	2.2	0.7	0.8	12	10	2
81_1597	*Cyphastrea salae*	5	2.2	1.8	0.6	0.5	11	10	2
81_1601	*Cyphastrea salae*	1	2.4	2.0	0.4	0.4	10	11	2
81_1601	*Cyphastrea salae*	2	2.4	1.7	0.4	1.0	10	8	2
81_1601	*Cyphastrea salae*	3	2.5	2.0	0.6	0.2	10	12	2
81_1601	*Cyphastrea salae*	4	2.5	1.9	0.4	0.2	10	14	2
81_1601	*Cyphastrea salae*	5	2.2	1.8	0.4	0.2	10	10	2
81_1625	*Cyphastrea salae*	1	2.2	1.7	0.5	0.7	12	8	2
81_1625	*Cyphastrea salae*	2	2.2	1.8	0.6	0.7	13	9	2
81_1625	*Cyphastrea salae*	3	2.5	1.9	0.6	1.2	13	11	2
81_1625	*Cyphastrea salae*	4	2.3	1.7	0.5	1.3	12	9	2
81_1625	*Cyphastrea salae*	5	2.0	1.7	0.5	0.4	11	10	2
81_1629	*Cyphastrea salae*	1	2.2	1.7	0.5	1.0	12	10	2
81_1629	*Cyphastrea salae*	2	2.0	1.7	1.0	1.4	12	9	2
81_1629	*Cyphastrea salae*	3	2.0	1.7	0.7	1.0	12	10	2
81_1629	*Cyphastrea salae*	4	2.2	1.9	0.6	0.8	12	9	2
81_1629	*Cyphastrea salae*	5	1.8	1.4	0.5	1.0	12	8	2
81_1638	*Cyphastrea salae*	1	2.7	2.0	0.7	1.2	12	8	2
81_1638	*Cyphastrea salae*	2	2.5	2.1	0.6	1.5	12	10	2
81_1638	*Cyphastrea salae*	3	2.6	1.8	0.7	1.2	12	10	2
81_1638	*Cyphastrea salae*	4	2.4	1.9	0.7	0.6	12	12	2
81_1638	*Cyphastrea salae*	5	2.0	1.5	0.6	0.5	10	13	2
81_1765	*Cyphastrea salae*	1	2.1	1.8	0.7	0.8	12	12	2
81_1765	*Cyphastrea salae*	2	2.0	1.5	0.6	1.2	12	14	2
81_1765	*Cyphastrea salae*	3	2.2	1.7	0.7	1.0	12	11	2
81_1765	*Cyphastrea salae*	4	1.8	1.5	0.6	1.2	12	14	2
81_1765	*Cyphastrea salae*	5	1.9	1.5	0.6	0.4	12	11	2
81_1822	*Cyphastrea salae*	1	2.2	1.6	0.4	1.0	12	6	2
81_1822	*Cyphastrea salae*	2	2.0	1.6	0.5	1.2	12	9	2
81_1822	*Cyphastrea salae*	3	2.0	1.6	0.5	0.9	11	7	2
81_1822	*Cyphastrea salae*	4	1.8	1.4	0.4	1.0	11	9	2
81_1822	*Cyphastrea salae*	5	1.8	1.5	0.4	1.2	12	7	2
81_4749	*Cyphastrea salae*	1	2.0	1.8	0.6	0.5	12	9	2
81_4749	*Cyphastrea salae*	2	1.7	1.5	0.5	0.4	12	8	2
81_4749	*Cyphastrea salae*	3	2.2	1.8	0.7	1.5	12	10	2
81_4749	*Cyphastrea salae*	4	1.8	1.5	0.6	1.2	12	7	2
81_4749	*Cyphastrea salae*	5	2.0	1.5	0.6	1.0	12	8	2

## References

[B1] Arrigoni R Berumen ML Huang D Terraneo TI Benzoni F (2017) Cyphastrea (Cnidaria: Scleractinia: Merulinidae) in the Red Sea: phylogeny and a new reef coral species. Invertebrate Systematics: in press.

[B2] Arrigoni R Berumen ML Chen CA Terraneo TI Baird AH Payri C Benzoni F (2016a) Species delimitation in the reef coral genera *Echinophyllia* and *Oxypora* (Scleractinia, Lobophylliidae) with a description of two new species. Molecular Phylogenetics and Evolution 105: 146–159. https://doi.org/10.1016/j.ympev.2016.08.023 27593164 10.1016/j.ympev.2016.08.023

[B3] Arrigoni R Benzoni F Huang D Fukami H Chen CA Berumen ML Hoogenboom M Thomson DP Hoeksema BW Budd AF Terraneo TI Kitano YF Baird AH (2016b) When forms meet genes: revision of the scleractinian genera *Micromussa* and *Homophyllia* (Lobophylliidae) with a description of two new species and one new genus. Contributions to Zoology 85: 387–422.

[B4] Ayre DJ Hughes TP (2004) Climate change, genotypic diversity and gene flow in reef-building corals. Ecology Letters 7: 273–278. https://doi.org/10.1111/j.1461-0248.2004.00585.x

[B5] Baird AH Cumbo VR Gudge S Keith SA Maynard JA Tan C-H Woolsey ES (2015) Coral reproduction on the world’s southernmost reef at Lord Howe Island, Australia. Aquatic Biology 23: 275–284. https://doi.org/10.3354/ab00627

[B6] Bouwmeester J Benzoni F Baird AH Berumen ML (2015) *Cyphastrea kausti* sp. n. (Cnidaria, Anthozoa, Scleractinia), a new species of reef coral from the Red Sea. ZooKeys 496: 1–13. https://doi.org/10.3897/zookeys.496.9433 10.3897/zookeys.496.9433 PMC4410153 25931952

[B7] Budd AF Stolarski J (2009) Searching for new morphological characters in the systematics of scleractinian reef corals: comparison of septal teeth and granules between Atlantic and Pacific Mussidae. Acta Zoologica 90: 142–165. https://doi.org/10.1111/j.1463-6395.2008.00345.x

[B8] Budd AF Stolarski J (2011) Corallite wall and septal microstructure in scleractinian reef corals: comparison of molecular clades within the family Faviidae. Journal of Morphology 272: 66–88. https://doi.org/10.1002/jmor.10899 21061280 10.1002/jmor.10899

[B9] Cairns SD (1999) Cnidaria Anthozoa: Deep-Water azooxanthellate Scleractinia from Vanuatu and Wallis and Futuna Islands. Mémoires du Muséum National d’Histoire Naturelle, Paris, 180: 31–167.

[B10] Cuif J-P Lecointre G Perrin C Tillier A Tillier S (2003) Patterns of septal biomineralization in Scleractinia compared with their 28S rRNA phylogeny: a dual approach for a new taxonomic framework. Zoologica Scripta 32: 459–473. https://doi.org/10.1046/j.1463-6409.2003.00133.x

[B11] Darriba D Taboada GL Doallo R Posada D (2012) jModelTest 2: more models, new heuristics and parallel computing. Nature Methods 9: 772 https://doi.org/10.1038/nmeth.2109 10.1038/nmeth.2109 PMC4594756 22847109

[B12] Ellis J Solander D (1786) The Natural History of many curious and uncommon Zoophytes, collected from various parts of the Globe. Systematically arranged and described by the late Daniel Solander. Benjamin White & Son, London, 206 pp [pls. 1–63] https://doi.org/10.5962/bhl.title.64985

[B13] Esper EJC (1793–1795) Die Planzenthiere: Fortsetzungen 1, Raspischen Buchhandlung, Nurnberg, 230 pp. [Abbildungen I: Madrepora pls. 1–87]

[B14] Francis MP (1993) Checklist of the coastal fishes of Lord Howe, Norfolk, and Kermadec Islands, Southwest Pacific Ocean. Pacific Science 47: 136–170.

[B15] Fukami H Chen CA Budd AF Collins AG Wallace CC Chuang Y-Y Dai C-F Iwao K Sheppard CRC Knowlton N (2008) Mitochondrial and nuclear genes suggest that stony corals are monophyletic but most families of stony corals are not (Order Scleractinia, Class Anthozoa, Phylum Cnidaria). PLoS ONE 3: e3222. https://doi.org/10.1371/journal.pone.0003222 10.1371/journal.pone.0003222 PMC2528942 18795098

[B16] Goloboff PA (1999) Analyzing large data sets in reasonable times: Solutions for composite optima. Cladistics 15: 415–428. https://doi.org/10.1111/j.1096-0031.1999.tb00278.x 10.1111/j.1096-0031.1999.tb00278.x 34902941

[B17] Goloboff PA Farris JS Nixon KC (2008) TNT, a free program for phylogenetic analysis. Cladistics 24: 774–786. https://doi.org/10.1111/j.1096-0031.2008.00217.x

[B18] Guindon S Gascuel O (2003) A simple, fast, and accurate algorithm to estimate large phylogenies by maximum likelihood. Systematic Biology 52: 696–704. https://doi.org/10.1080/10635150390235520 14530136 10.1080/10635150390235520

[B19] Harriott VJ Smith SDA Harrison PL (1994) Patterns of coral community structure of subtropical reefs in the Solitary-Islands Marine Reserve, eastern Australia. Marine Ecology Progress Series 109: 67–76. https://doi.org/10.3354/meps109067

[B20] Harriott VJ Harrison PL Banks SA (1995) The coral communities of Lord Howe Island. Marine and Freshwater Research 46: 457–465. https://doi.org/10.1071/MF9950457

[B21] Hoeksema BW (2015) *Cyphastrea* Milne Edwards & Haime 1848. Accessed through: World Register of Marine Species at http://www.marinespecies.org/aphia.php?p=taxdetails&id=206488 [on 2017-01-25]

[B22] Huang D (2012) Threatened reef corals of the world PLoS ONE 7: e34459. https://doi.org/10.1371/journal.pone.0034459 10.1371/journal.pone.0034459 PMC3316686 22479633

[B23] Huang D Roy K (2013) Anthropogenic extinction threats and future loss of evolutionary history in reef corals. Ecology and Evolution 3: 1184–1193. https://doi.org/10.1002/ece3.527 23762506 10.1002/ece3.527 PMC3678474

[B24] Huang D Roy K (2015) The future of evolutionary diversity in reef corals. Philosophical Transactions of the Royal Society B-Biological Sciences 370: 20140010. https://doi.org/10.1098/rstb.2014.0010 10.1098/rstb.2014.0010 PMC4290424 25561671

[B25] Huang D Licuanan WY Baird AH Fukami H (2011) Cleaning up the ‘Bigmessidae’: Molecular phylogeny of scleractinian corals from Faviidae, Merulinidae, Pectiniidae and Trachyphylliidae. BMC Evolutionary Biology 11: 37 https://doi.org/10.1186/1471-2148-11–37 21299898 10.1186/1471-2148-11-37 PMC3042006

[B26] Huang D Benzoni F Fukami H Knowlton N Smith ND Budd AF (2014) Taxonomic classification of the reef coral families Merulinidae, Montastraeidae, and Diploastraeidae (Cnidaria: Anthozoa: Scleractinia). Zoological Journal of the Linnean Society 171: 277–355. https://doi.org/10.1111/zoj.12140

[B27] Huelsenbeck JP Ronquist F (2001) MRBAYES: Bayesian inference of phylogenetic trees. Bioinformatics 17: 754–755. https://doi.org/10.1093/bioinformatics/17.8.754 11524383 10.1093/bioinformatics/17.8.754

[B28] Katoh K Standley DM (2013) MAFFT multiple sequence alignment software version 7: improvements in performance and usability. Molecular Biology and Evolution 30: 772–780. https://doi.org/10.1093/molbev/mst010 23329690 10.1093/molbev/mst010 PMC3603318

[B29] Katoh K Toh H (2008) Recent developments in the MAFFT multiple sequence alignment program. Briefings in Bioinformatics 9: 286–298. https://doi.org/10.1093/bib/bbn013 18372315 10.1093/bib/bbn013

[B30] Katoh K Misawa K Kuma K Miyata T (2002) MAFFT: a novel method for rapid multiple sequence alignment based on fast Fourier transform. Nucleic Acids Research 30: 3059–3066. https://doi.org/10.1093/nar/gkf436 12136088 10.1093/nar/gkf436 PMC135756

[B31] Katoh K Asimenos G Toh H (2009) Multiple alignment of DNA sequences with MAFFT. In: Posada D (Ed.) Bioinformatics for DNA Sequence Analysis. Humana Press, New York, 39–63. https://doi.org/10.1007/978-1-59745-251-9_3 10.1007/978-1-59745-251-9_3 19378139

[B32] Kitahara MV Cairns SD Stolarski J Blair D Miller DJ (2010) A comprehensive phylogenetic analysis of the Scleractinia (Cnidaria, Anthozoa) based on mitochondrial CO1 sequence data. PLoS ONE 5: e11490. https://doi.org/10.1371/journal.pone.0011490 10.1371/journal.pone.0011490 PMC2900217 20628613

[B33] Kitahara MV Fukami H Benzoni F Huang D (2016) The new systematics of Scleractinia: integrating molecular and morphological evidence. In: Goffredo S Dubinsky Z (Eds) The Cnidaria, Past, Present and Future. Springer International Publishing, Cham, 41–59. https://doi.org/10.1007/978-3-319-31305-4_4

[B34] Lamarck JB (1801) Système des animaux sans vertèbres, ou tableau général des classes, des ordres et des genres de ces animaux; Présentant leurs caractères essentiels et leur distribution, d’après la considération de leurs rapports naturelles et de leur organisation, et suivant l’arrangement établi dans les galeries du Muséum d’Histoire Naturelle, parmi leurs dépouilles conservées; Précédé du discours d’ouverture du Cours de Zoologie, donné dans le Muséum National d’Histoire Naturelle l’an 8 de la République. Published by the authour and Deterville, Paris, 432 pp.

[B35] Lamarck JBM de (1816) Histoire naturelle des animaux sans vertèbres. Tome deuxième. Paris, Verdière, 568 pp.

[B36] Milne Edwards M Haime J (1848) Recherches sur les polypiers, 4^eme^ mémoire. Monographie des Astréides. Annales des Sciences Naturelles 10: 209–320.

[B37] Mizerek TL Baird AH Beaumont LJ Madin JS (2016) Environmental tolerance governs the presence of reef corals at latitudes beyond reef growth. Global Ecology and Biogeography 25: 979–987. https://doi.org/10.1111/geb.12459

[B38] Moll H Best MB (1984) New scleractinian corals (Anthozoa: Scleractinia) from the Spermonde Archipelago, South Sulawesi, Indonesia. Zoologische Mededelingen 58: 47–58.

[B39] MPA (2010) Lord Howe Island Marine Park: zoning plan review report, NSW Department of Environment and Climate Change, Sydney, 132 pp.

[B40] Nemenzo F (1959) Systematic studies on Philippine shallow water scleractinians: II. Suborder Faviida. Natural and Applied Science Bulletin, University of the Philippines 16: 73–135. [pls. 1–24]

[B41] Nixon KC (1999) The Parsimony Ratchet, a new method for rapid parsimony analysis. Cladistics 15: 407–414. https://doi.org/10.1111/j.1096-0031.1999.tb00277.x 10.1111/j.1096-0031.1999.tb00277.x 34902938

[B42] Noreen AME Harrison PL Van Oppen MJH (2009) Genetic diversity and connectivity in a brooding reef coral at the limit of its distribution. Proceedings of the Royal Society B-Biological Sciences 276: 3927–3935. https://doi.org/10.1098/rspb.2009.1050 10.1098/rspb.2009.1050 PMC2825775 19710055

[B43] Posada D (2008) jModelTest: Phylogenetic model averaging. Molecular Biology and Evolution 25: 1253–1256. https://doi.org/10.1093/molbev/msn083 18397919 10.1093/molbev/msn083

[B44] Rambaut A Suchard MA Xie D Drummond AJ (2014) Tracer: MCMC Trace Analysis Tool. Version 1.6. Available from: http://beast.bio.ed.ac.uk/Tracer

[B45] Ronquist F Huelsenbeck JP (2003) MrBayes 3: Bayesian phylogenetic inference under mixed models. Bioinformatics 19: 1572–1574. https://doi.org/10.1093/bioinformatics/btg180 12912839 10.1093/bioinformatics/btg180

[B46] Ronquist F Teslenko M van der Mark P Ayres DL Darling A Höhna S Larget B Liu L Suchard MA Huelsenbeck JP (2012) MrBayes 3.2: Efficient Bayesian phylogenetic inference and model choice across a large model space. Systematic Biology 61: 539–542. https://doi.org/10.1093/sysbio/sys029 22357727 10.1093/sysbio/sys029 PMC3329765

[B47] Schmidt-Roach S Miller KJ Andreakis N (2013) *Pocillopora aliciae*: a new species of scleractinian coral (Scleractinia, Pocilloporidae) from subtropical Eastern Australia. Zootaxa 3626: 576–582. https://doi.org/10.11646/zootaxa.3626.4.11 26176158 10.11646/zootaxa.3626.4.11

[B48] Stamatakis A (2006) RAxML-VI-HPC: Maximum likelihood-based phylogenetic analyses with thousands of taxa and mixed models. Bioinformatics 22: 2688–2690. https://doi.org/10.1093/bioinformatics/btl446 16928733 10.1093/bioinformatics/btl446

[B49] Stamatakis A Hoover P Rougemont J (2008) A rapid bootstrap algorithm for the RAxML web servers. Systematic Biology 57: 758–771. https://doi.org/10.1080/10635150802429642 18853362 10.1080/10635150802429642

[B50] Stolarski J Roniewicz E (2001) Towards a new synthesis of evolutionary relationships and classification of Scleractinia. Journal of Paleontology 75: 1090–1108. doi 10.1017/S0022336000017157

[B51] Terraneo TI Benzoni F Arrigoni R Berumen ML (2016) Species delimitation in the coral genus *Goniopora* (Scleractinia, Poritidae) from the Saudi Arabian Red Sea. Molecular Phylogenetics and Evolution 102: 278–294. https://doi.org/10.1016/j.ympev.2016.06.003 27321092 10.1016/j.ympev.2016.06.003

[B52] Veron JEN (1974) Southern geographic limits to the distribution of Great Barrier Reef hermatypic corals. Proceedings of the 2^nd^ International Coral Reef Symposium 1: 465–473.

[B53] Veron JEN How RA Done TJ Zell LD Dodkin MJ O’Farrell AF (1974) Corals of the Solitary Islands, Central New South Wales. Australian Journal of Marine and Freshwater Research 25: 193–208. https://doi.org/10.1071/MF9740193

[B54] Veron JEN (1993) A biogeographic database of hermatypic corals. Australian Institute of Marine Science Monograph Series 10: 1–433.

[B55] Veron JEN (1995) Corals in space and time. Southwood Press, Sydney, 321 pp.

[B56] Veron JEN Done TJ (1979) Corals and coral communities of Lord Howe Island. Australian Journal of Marine and Freshwater Research 30: 203–236. https://doi.org/10.1071/MF9790203

[B57] Verrill AE (1865) Classification of polyps (extract condensed from Synopsis of the Polyps and Corals of the North Pacific Exploring Expedition under Commodore C. Ringgold and Captain John Rodgers, U.S.N.). Communications of the Essex Institute 4: 145–152.

